# Investigating Risk of Suboptimal Macro and Micronutrient Intake and Their Determinants in Older Danish Adults with Specific Focus on Protein Intake—A Cross-Sectional Study

**DOI:** 10.3390/nu11040795

**Published:** 2019-04-06

**Authors:** Simon Rønnow Schacht, Mads Vendelbo Lind, Rasmus Leidesdorff Bechshøft, Grith Højfeldt, Søren Reitelseder, Tenna Jensen, Astrid Pernille Jespersen, Dennis Sandris Nielsen, Lars Holm, Inge Tetens

**Affiliations:** 1 Vitality—Centre for Good Older Lives, Department of Nutrition, Exercise and Sports, University of Copenhagen, 1958 Frederiksberg C, Denmark; madslind@nexs.ku.dk (M.V.L.); ite@nexs.ku.dk (I.T.); 2 Institute of Sports Medicine Copenhagen, Department of Orthopaedic Surgery M, Bispebjerg Hospital, Copenhagen, Denmark & Department of Biomedical Sciences, Faculty of Health and Medical Sciences, University of Copenhagen, 2200 Copenhagen, Denmark; r.bechshoeft@gmail.com (R.L.B.); grith.westergaard.hoejfeldt.01@regionh.dk (G.H.); s.reitelseder@gmail.com (S.R.); larsh@sund.ku.dk (L.H.); 3 CoRe—Copenhagen Centre for Health Research in the Humanities, Saxo Institute, Faculty of Humanities, University of Copenhagen, 2300 Copenhagen, Denmark; tennaje@hum.ku.dk (T.J.); apj@hum.ku.dk (A.P.J.); 4 Department of Food Science, University of Copenhagen, 1958 Frederiksberg C, Denmark; dn@food.ku.dk; 5 School of Sport, Exercise and Rehabilitation Sciences, University of Birmingham, Birmingham B15 2TT, UK

**Keywords:** Dietary Reference Values, cut-point method, nutrient adequacy, nutrient deficiency, protein deficiency, nutrient determinants, elderly, sarcopenia, bone mineral density, Counteracting Age-related Loss of Skeletal Muscle Mass (CALM) study

## Abstract

Suboptimal intake of nutrients is associated with adverse health outcomes. The current study investigated the risk of suboptimal macro and micronutrient intake and their potential determinants in a cross-sectional study of community-dwelling older Danish adults (65–81 years). Nutrient intake was obtained through a 3-day weighted dietary record and information on personal characteristics and attitudes towards specific foods and dietary habits and nutrition through questionnaires. Dietary Reference Values (DRV) from the Nordic Nutrition Recommendations were used for the assessment. Among 157 participants, 68% and 66% had risk of suboptimal intake of dietary fiber and saturated fatty acids (SFA). For mono-unsaturated fatty acids (MUFA) and poly-unsaturated fatty acids (PUFA), the numbers were 47% and 62%, respectively. Increased risk of suboptimal protein intake was estimated in 3 to 45% of the participants, depending on the criteria used for the DRV and of the mode of expressing protein intake. Fifty percent had intakes of alcohol above the maximum recommended intake. Risk of micronutrient inadequacy was particularly high for vitamin D and thiamine (80 and 45%, respectively). Total energy intake and attitude regarding healthy eating were associated with lower nutrient intake. The current study illustrates that there is room for improvements in the dietary quality of community dwelling older Danish adults.

## 1. Introduction

Within the next two to three decades, older adults, defined as ≥65 years of age are projected to increase with up to 100%, globally [1]. These demographic changes give rise to increasing concerns regarding both the individual as well as societal challenges that may follow. Diet and nutritional status are recognized as important determinants for maintenance of health and functionality throughout old age [2]. Observational studies have shown that low intakes of micronutrients are associated with increased risk of frailty in older individuals [3] as well as increased difficulties with independent living [4]. However, maintaining proper nutritional status throughout old age can be challenging for several reasons. These include, for instance, social deprivation, loss of appetite, decreased absorptive capacity, and deteriorating oral health [5,6]. The nutritional status of the older population is therefore a growing public health concern. 

A common nutritional issue in older individuals is a low energy intake, affecting both macro- and micronutrient intakes. Protein adequacy is another nutritional issue and protein intake is considered a primary dietary determinant of age-related loss of muscle mass and strength (sarcopenia) [7]. Both total protein intake and protein distribution throughout the day are potentially important factors in the development of sarcopenia [7]. If left unnoticed, sarcopenia can increase the risk of several age-related diseases, care dependency and lower the quality of life [8,9]. 

It has been reported that 3 to 12% of older individuals in Western countries have protein intakes below the estimated average requirement [10,11], which entails a high risk of nutrient inadequacy [12]. Based on nitrogen balance studies, the estimated average requirements for adults have been set at 0.66 g protein per kilogram of bodyweight (g/kg BW) corresponding to a recommended daily intake of 0.83 g/kg BW high quality protein, theoretically satisfying an adequate intake for 97.5% of the adult population [13]. However, these data stems primarily from studies investigating healthy younger adults. Emerging evidence from prospective cohort studies indicates that the protein intake required to support body protein turnover and to counteract development of sarcopenia is considerably higher [14,15]. Based on this evidence, the Recommended Intake Range in the recent version of the Nordic Nutrition Recommendations (NNR) was set to 1.1–1.3 g/kg BW (15–20 E%) for individuals >65 years of age [12]. Assessing the risk of suboptimal protein intake in older individuals is thus challenging as cut-off values can be derived from either nitrogen balance studies or studies investigating risk of sarcopenia.

Inadequate intake of micronutrients is also of concern in many older population groups. A systematic review of micronutrient intake and potential inadequacies in community-dwelling older adults living in Western countries reported that risk of inadequacy was common for six micronutrients, in particular: vitamin D, vitamin B1 (thiamine), vitamin B2 (riboflavin), calcium, magnesium, and selenium [4]. 

The aim of the present study was to assess the proportion of individuals in a group of apparently healthy older Danes with macronutrient intakes not meeting dietary reference values and to investigate differences in the assessed proportion of individuals not meeting protein recommendations when applying different existing cut-off values. Additionally, the study investigated risk of inadequate micronutrient intakes as well as potential determinants of low nutrient intake. 

## 2. Materials and Methods

Cross-sectional baseline data from the ‘Counteracting Age-related Loss of Skeletal Muscle Mass’ (CALM) study were used in the current study. The CALM study is a randomized intervention trial investigating dietary and physical activity strategies to prevent sarcopenic progression in healthy and independently living individuals 65 years of age or above. The intervention trial is described in detail by Bechshøft et al. (2016) [16]. 

### 2.1. Study Population

Study participants were recruited within the greater Copenhagen area via local newspapers, flyers, radio programs, social media and presentations at senior centers and public events. Inclusion criteria were: healthy, independently living individuals (≥65 years of age). Exclusion criteria were: care dependency, dementia, heavy alcohol consumption, >1 h of weekly heavy strength training, no Danish citizenship as well as specific diseases that could interfere with participation [16]. Altogether, 205 elderly Danish men and women were included in the CALM study. Of these, 184 study participants (53.3% men) completed the baseline dietary registration and were included in the present study. 

### 2.2. Ethical Approval and Consent to Participate

The Danish Regional Ethical Committees of the Capital Region approved the study (J-nr. H-4-2013-070). All participants gave written informed consent in accordance with the Declaration of Helsinki II. The CALM study is registered at ClinicalTrials.gov; Identifier: NCT02034760.

### 2.3. Dietary Assessment

Following individual instructions from staff, participants weighed their dietary intake for three consecutive days (Wednesday to Friday) and entered the information into food records. Trained staff entered the completed dietary records into the electronic dietary assessment tool, VITAKOST™ (MADLOG ApS, Kolding, Denmark) for estimation of study participants’ individual daily nutrient intake. Nutrient intake was calculated for each individual using the Danish Food Composition Databank (version 7.01; Søborg; Denmark). Sugar intake was calculated as total free sugar (intrinsic and added). For protein, the distribution of intake throughout the day was assessed at breakfast, lunch, dinner and in-between meals, respectively. Nutrient intake was assessed from foods only.

### 2.4. Under-Reporters

Potential under-reporters were identified and excluded from the analyses. Under-reporters were defined according to the ratio of reported mean energy intake to basal metabolic rate (EI:BMR) as described by Black [17] using an EI:BMR ≤ 1.0 as a cut-off point and a PAL of 1.5. BMRs were estimated according to age and sex as described by Black.

### 2.5. Qualitative Questionnaire

At baseline, study participants received an extensive questionnaire containing both closed and open questions aiming to provide insights into participants’ food perceptions and habits and past dietary changes. The questionnaire consisted of four sections: (1) marital status, lifestyle and socio-economic factors; (2) food preferences, and attitude towards food and nutrition; (3) dietary changes in the past, and (4) perceptions of protein and their preferences for protein rich food types.

### 2.6. Analyses and Statistical Methods

Descriptive statistics are presented as medians with the respective 5th and 95th percentiles. Percentage of individuals not meeting macro- and micronutrient reference values was estimated using the Dietary Reference Values of the NNR [12], which uses the terms Average Requirement (AR), Reference Intake (RI), and Recommended Intake Range (RIR). 

The risk of suboptimal macronutrient intakes was estimated as the percentage of individuals below or above the RIR bounds for carbohydrates, total fat, mono-unsaturated fatty acids (MUFA), and poly-unsaturated fatty acids (PUFA). The risk of suboptimal intake of dietary fiber was estimated as the percentage of individuals below the minimum RI value and for saturated fat and alcohol as the percentage of individuals above the maximum RI value [12]. 

For protein intake, we estimated the percentage of individuals with intakes below the suggested AR of 0.66 g/kg BW. Additionally, we estimated the percentage of individuals with intakes outside the NNR protein recommendations given both as an energy percentage (RIR: 15–20 E%) and as gram per kg body weight (RIR: 1.1–1.3 g/kg BW). 

Risk of micronutrient inadequacy was assessed by the “cut-point-method” [18] where the percentage of individuals with intakes below the established AR was assessed as being at *risk of inadequacy*. For nutrients with no established AR, we used the term *risk of suboptimal intake* to indicate that risk might occur, but with a higher level of uncertainty. 

Potential determinants of nutrient intake was assessed by stratifying variables of interest (‘age’, ‘living with a partner’, ‘attitude towards healthy eating’, and ‘total energy intake’) and comparing the risk of suboptimal intake of protein and dietary fiber as well as risk of nutrient inadequacy of vitamin D, thiamine, selenium, and iodine between these strata. The influence of age was assessed by comparing the youngest third of the population versus the oldest third of the population. The influence of living with a partner was assessed by comparing individuals living alone to individuals living with a partner. Attitude towards healthy eating was assessed by constructing a “healthy attitude index”. This index was based on a 1–5 scoring system on four separate statements relating to a healthy attitude towards eating: “It is important for me to eat healthy”, “It is important that my food contains a lot of vitamins and minerals”, “I try to follow the official dietary guidelines”, and “I eat whatever I feel like without thinking about whether it is healthy or not”. Scoring on the last statement was inversed, as this was the only statement where lower scoring indicated a ‘healthier’ attitude. A comparison was made between half of the population scoring lowest and half of the population scoring highest. The influence of total energy intake (relative to bodyweight) was estimated by comparing half of the population with the lowest energy intake to half of the population with the highest energy intake. Potential differences between groups were estimated based on the chi-squared statistics. 

Statistical significance was considered as *p* < 0.05. All analyses were performed in R, version 3.5.1 (R Core Team (2018). R: A language and environment for statistical computing. R Foundation for Statistical Computing, Vienna, Austria. URL https://www.R-project.org/).

## 3. Results

### 3.1. Participant Characteristics

Of the 184 participants included in the study, 27 individuals (14.7%) were categorized as under-reporters and excluded from our analyses. Baseline characteristics are shown for the remaining 157 study participants (50.3% males) in Table 1. 

### 3.2. Intake of Macronutrients and Alcohol 

The median intake of recorded macro- and micronutrients are shown for male and female study participants in Table 2. 

### 3.3. Comparing Macronutrient Intake to the Recommend Intake Values 

#### 3.3.1. Protein 

**Figure 1:**
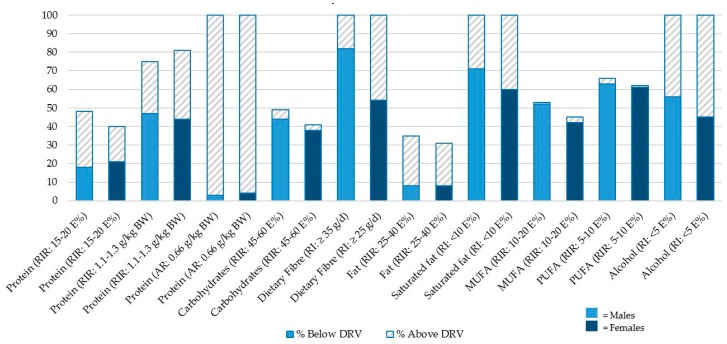
Percentage of study participants with intakes of macronutrients, dietary fiber and alcohol below and above respective DRVs. For nutrients with DRVs given as intervals, the figure shows the percentage of individuals below the lower bound and above the higher bound, respectively. The remaining percentages represent intakes within the reference interval (not indicated by bars). For nutrients with DRVs given as a single cut-off value, the figure shows the percentage of individuals below and above this value. PUFA: poly-unsaturated fatty acids; MUFA: mono-unsaturated fatty acids.

Using the RIR as criteria, 18% of men and 21% of women in our study population had protein intakes below the NNR’s lower RIR bound of 15 E% whereas 30% of men and 19% of women had intakes above the upper RIR bound of 20 E% (Table 3 & Figure 1). When applying the lower RIR bound of 1.1 g protein per kg BW as the cut-off, 47% of men and 44% of women fell below this value. Only 3% of men and 4% women had intakes below the AR of 0.66 g/kg BW.

The present study also examined the distribution of protein intake at different meals throughout the day in the study population. The mean protein intake was 20.4% and 20.2% of total daily protein intake at breakfast, 27.1% and 23.2% at lunch and 40.3% and 42.1% at dinner for men and women, respectively. Mean protein intake in-between meals was 12.2% of the total daily protein intake for men and 14.5% for women.

#### 3.3.2. Carbohydrate and Dietary Fiber

Forty four percent of men and 38% of women had carbohydrate intakes below the lower RIR bound of 45 E%, and 5% and 3% had intakes above the upper RIR bound of 60 E%, respectively. The proportion of individuals with dietary fiber intake below recommended values was 82% of men and 54% of women.

#### 3.3.3. Fat

Eight percent of both men and women had total fat intakes below the lower RIR bound of 25 E%, and 27% and 23% had intakes above the upper RIR bound of 40 E%, respectively. The proportion of individuals with SFA intakes above the RI cut-off of 10 E% was 71% of men and 60% of women. The proportion of individuals with a MUFA intake below the lower RIR bound of 10 E% was 52% of men and 42% of women, and 63% of men and 61% of women had PUFA intakes below the lower RIR bound of 5 E%, respectively.

#### 3.3.4. Alcohol 

Fifty six percent of men and 45% of women had alcohol intakes above the RI value of 5 E%.

### 3.4. Comparing Micronutrient Intake to the Estimated Average Requirements

#### 3.4.1. Vitamins

Risk of inadequate intake was above 30% for both sexes for two of the vitamins. For vitamin D, 76% of men and 83% of women had intakes below the AR of 7.5 µg/day. For thiamine, 57% of men and 33% of women had intakes below the ARs of 1.2 mg/day and 0.9 mg/day, respectively. Risk of inadequacy for these nutrients was therefore evaluated as high in the current population of older community-dwelling Danes.

Thirty three percent of men and 27% of women had vitamin A intakes below the AR of 600 RE/day and 500 RE/day, respectively. 38% of men and 26% of women had riboflavin intakes below the AR of 1.4 mg/day and 1.1 mg/day, respectively. Forty six percent of men and 18% percent of women had intakes of vitamin E below the AR of 6 α-TE/day and 5 α-TE/day, respectively. Risk of inadequacy for these nutrients were evaluated as moderate in the current study population.

#### 3.4.2. Minerals and Trace Elements 

The proportion of individuals at risk of inadequate intake of minerals and trace elements was below 30% for both sexes for all of the assessed minerals or trace elements (Table 4). 28% of men and 46% of women had iodine intakes below the AR of 100 µg/day, and 25% of men and 33% of women had selenium intakes below the AR of 35 mg/day and 30 mg/day, respectively. For iron, 16% of men and 13% of women had intakes below the AR of 7 mg/day and 6 mg/day, respectively. For the remaining minerals and trace elements, the percentage of men and women below the respective ARs were all <12%.

### 3.5. Potential Determinants of Low Nutrient Intake

The proportion of individuals with intakes below either recommended intake values or ARs for six selected nutrients stratified by potential determinants is presented in Table 5. Study participants with a healthy attitude towards eating had significantly lower risk of inadequacy of vitamin D and selenium when compared to participants with a ‘non-healthy’ attitude. Having a low energy intake (relative to bodyweight) was associated with lower intakes of both protein and dietary fibers. No statistical differences were observed when comparing “youngest” versus “oldest” individuals or people living alone versus people living with a partner.

## 4. Discussion

In the present study, we found that a substantial proportion of this group of older community-dwelling Danes were at risk of having suboptimal or inadequate nutrient intakes. We further observed large differences in the protein risk assessment depending on whether the chosen cut-off value was obtained from nitrogen balance studies or studies investigating risk of sarcopenia and other muscle or strength related outcomes. 

### 4.1. Protein Intake and Distribution

Assessing risk of adequate protein intake in older adults is currently challenging as recent evidence suggests that other criteria for the DRV may be pertinent. Based on the collective evidence from nitrogen balance studies [13], nutrition and health institutes such as EFSA and the U.S National Academy of Medicine (formerly the IOM) use the EAR of 0.66 g/kg BW as the criteria for setting an adequate protein intake of 0.8 g/kg BW for all age groups, including older subjects. Yet, mounting evidence indicates that increasing protein intake to 1.1 g/kg BW or higher may have additional health benefits in relation to the maintenance of muscle mass and reducing the rate of sarcopenic development [19,20,21]. As shown in Table 3, the proportion of older subjects in our study population with a protein intake below the reference value lower threshold was considerably higher when the reference values were obtained from studies investigating risk of sarcopenia compared to nitrogen balance studies. These results highlight the importance of the chosen criteria when risk of inadequate or suboptimal protein intake is determined in older adults. As the risk assessment further varied depending on the reference values being calculated as E% or as g/kg BW, future studies reporting on or comparing protein intake in older study populations should be highly aware of the methodology and criteria applied.

In addition to the total intake of protein, the distribution of protein throughout the day has been suggested to be of relevance with regards to increasing overall protein intake in older people [22] as well as optimizing muscle protein synthesis [7]. The latter, however, remains an open question and the importance of daily protein distribution likely depends on the total daily amount being consumed [23,24]. In the present study, the estimated protein intake was highest at dinnertime, lower at lunch and lowest at breakfast and in-between meals, for both sexes. Hence, if additional protein is desired, this suggests room for an increased intake around breakfast and in-between meals in community-dwelling older adults.

### 4.2. Carbohydrate, Fat, and Alcohol

The recommended intake of dietary fibers was not met in 68% of our study population. Consumption of adequate dietary fiber is associated with numerous health benefits such as lowered risk of obesity, CVD, type-2-diabetes and certain cancers [25]. Furthermore, consuming dietary fiber might be of particular importance in elderly individuals as fiber can assist proper gut health and alleviate common gastrointestinal symptoms such as constipation [26]. Over 65% of the study population consumed SFA in amounts exceeding the recommendations and 50–60% did not reach recommendations for MUFA and PUFA. Although recent evidence is conflicting with regards to the cardiovascular health implications of dietary fat quality [27], more studies are needed before the scientific evidence is sufficient to change current dietary recommendations. In the present study, half of the study population exceeded the upper limit for the recommended alcohol consumption of 5 E%. The remaining half of the population was split into two groups: those that had alcohol intakes below 5 E% (33%) and those that did not consume alcohol (17%). Although low and moderate alcohol intake may have positive effects on stress relief, mood and social life in older people [28], exceeding alcohol recommendations is a risk factor for several age-related diseases such as liver cirrhosis [29] and multiple forms of cancer [30]. 

### 4.3. Micronutrients

The risk of micronutrient inadequacy was relatively high for several micronutrients. As suggested in the systematic review by Borg et al. (2015) [4], specific micronutrients were assessed as being a serious nutritious concern if more than 30% of both men and women had intakes below the respective AR. In the present study, this was true for two nutrients: vitamin D and thiamine. Adequate amounts of vitamin D is important for bone health [31] and potentially also for musculoskeletal [32] and cognitive functions [33] as well as a range of additional diseases [34]. Vitamin D can be provided via sun exposure, yet, the suboptimal 25-hydroxyvitamin D blood concentrations observed in several older populations indicate that dietary vitamin D is important [35,36]. Thiamine insufficiency may also pose a health concern for older individuals and subclinical deficiencies are perhaps relatively common, particularly in older populations [37], where it potentially increases fatigue as well as the risk of cardiac and cognitive impairments [37]. For several nutrients, the proportion of individuals with inadequate intakes was less than 30% in both sexes, but still relatively prevalent. This was true for vitamin A, and selenium (20–30% inadequacy in both men and women) as well as for riboflavin, vitamin E and iodine (>30% inadequacy for only one of the sexes).

Assessments of micronutrient inadequacy should preferably be estimated over longer durations. This is particularly true for nutrients such as vitamin A and C [38]. In spite of this, the current results are likely valid as these were relatively similar to those presented in a systematic review investigating nutrient inadequacy in Western elderly [4]. In this review, 30–40% of older western men and women were estimated at risk of inadequate intakes of riboflavin and selenium. For vitamin A, this was true for 25–30%. Risk of iodine inadequacies was reported to be less pronounced compared with the current study of elderly Danes (20–26% vs 27–42%). Risk of vitamin E inadequacies was reported to be lower for western men compared to Danish men (26% vs. 46%), but similar for women. The present study did not find risk of calcium inadequacy to be a serious concern, whereas this was reported for western elderly in general. 

Vitamin and mineral supplementation is commonly consumed in the Danish population, particularly in the youngest and oldest individuals [39]. More than 60% of men and 80% of women in the Danish older population have been reported to consume dietary supplements, with the most common form of supplementation being multivitamins, fish oil, calcium, and vitamin D [40]. As dietary supplementation was not included in our analyses, the risk of micronutrient inadequacy is likely overestimated in the present study. Nonetheless, investigating the nutrient contribution from food sources alone provides valuable information regarding the quality of diet in this population group. 

### 4.4. Determinants of Inadequate Nutrient Intake

We found that a lower energy intake was associated with higher risk of suboptimal nutrient intake, which highlights the importance for older individuals to consume adequate amounts of energy in order to fulfill requirements of all nutrients from foods. Additionally, caring about the nutrient quality of the diet and eating according to official dietary guidelines (‘healthy attitude towards eating’) was associated with having lower risk of suboptimal nutrient intake (Table 5). Age and living alone were not associated with nutrient intake in the present study. This could be explained by the fact that this was a well-functioning study population with a relatively narrow age spread. 

### 4.5. Strengths and Limitations

A strength of the present study is the measurement of both quantitative and qualitative information relating to the study participants’ dietary consumption, allowing informative analyses such as the assessment of potential determinants of nutrient intake.

The main limitation of the present study is the relatively small sample size. The utilization of a weighed dietary record was selected to get an insight into the timing of dietary intake with the tradeoff that the recording was continued for only three days. Despite these potential concerns, the median intake of most of the estimated nutrients in the present study are comparable to those reported for a similar age group in the Danish national dietary survey, which used a more comprehensive seven days dietary recording [41]. Also, our findings on the prevalence of risk of micronutrient inadequacies in Danish elderly are relatively similar to the estimated risk of inadequacy observed for Western elderly in general [4]. Therefore, we trust that the present results are likely to represent valid dietary data. As heavy alcohol consumption was included as one of the exclusion criteria, the present study potentially underestimates the average alcohol intake. Data was only available on total sugar intake (added and intrinsic sugar combined). Therefore, an estimation of the percentage of individuals with added sugar intakes higher than the DRV of ≥10 E% was not possible. Lastly, it is important to acknowledge that true nutrient inadequacy cannot be assessed based on dietary data only. This could only be done by also including relevant biomarkers of nutrient status.

## 5. Conclusions

In a group of apparently healthy community-dwelling older Danes we found that intakes of saturated fats and alcohol were too high compared to official dietary reference values. In contrast, the intakes of MUFA, PUFA and dietary fiber were too low. Assessing the risk of suboptimal protein intake is currently difficult in older individuals as this assessment depends on whether the reference values are based on nitrogen balance studies or studies considering risk of sarcopenia. We found a high prevalence of subjects at risk of vitamin D and thiamine inadequacy. Risk of inadequacy was less profound for vitamin A, riboflavin, vitamin E, iodine, and selenium, but could still be considered potentially problematic. Consuming adequate amounts of energy as well as having a healthy attitude towards eating were identified as potentially determinants of nutrient adequacy in the current study. The present results on specific nutrients of concern in this group of community-dwelling older adults and the potential determinants influencing nutrient intake can be used for designing more targeted intervention studies in this segment of the population.

## Figures and Tables

**Table 1:** Baseline characteristics for the study population (values are presented as medians and (P5–P95)).

	Men (*n* = 79)	Women (*n* = 78)
Age, years	69 (65–77)	70 (65–78)
Weight, kg	78.9 (64.6–100.4)	66.7 (54.3–92.5)
Height, m	1.76 (1.67–1.87)	1.66 (1.57–1.79)
Body mass index (BMI), kg/m^2^	25.3 (21.5–32.7)	23.9 (19.5–32.2)
Waist circumference, cm	94 (82–115)	85 (71–111)
Hip circumference, cm	98 (91–109)	101 (91–116)
Systolic blood pressure, mmHg	142 (116–173)	140 (113–167)
Diastolic blood pressure, mmHg	84 (70–100)	81 (68–96)
400 m walking time, seconds	223 (191–286)	243 (213–303)
Married/cohabitating %	83	46

**Table 2:** Daily intake of nutrients in the ‘Counteracting Age-related Loss of Skeletal Muscle Mass’ (CALM) population (values are presented as medians and (P5–P95)).

	Men (*n* = 79)	Women (*n* = 78)
Energy, MJ/day	9.2 (6.9–12.4)	7.9 (5.4–10.5)
Protein, E%	17.9 (13.7–24.2)	17.7 (13.1–24.8)
Protein, g/day	92.3 (60.8–129.0)	79.4 (48.8–114.5)
Protein, g/kg bodyweight	1.1 (0.8–1.7)	1.2 (0.7–1.8)
Carbohydrates, E%	46.1 (35.7–59.9)	46.3 (36.0–56.5)
Sugar, E%	13.8 (8.2–23.0)	16.9 (8.3–26.7)
Dietary fiber, g/day	23.9 (13.0–40.4)	23.9 (15.3–38.0)
Fat, E%	35.6 (24.7–47.5)	35.8 (24.0–44.5)
Saturated fat, E%	12.0 (6.9–19.6)	11.3 (6.9–16.4)
Mono-unsaturated fat, E%	9.9 (5.5–16.6)	10.6 (5.1–17.0)
Poly-unsaturated fat, E%	4.3 (1.7–7.7)	4.6 (2.5–7.5)
Alcohol, E%	5.7 (0.0–23.2)	4.5 (0.0–13.8)
Vitamin A, RE	830 (209–3167)	767 (239–2441)
Vitamin D, µg	4.3 (1.0–21.6)	3.3 (0.7–15.3)
Vitamin E, α-TE	6.5 (2.2–12.1)	6.8 (2.9–16.8)
Thiamine, mg	1.2 (0.7–2.0)	1.0 (0.6–1.7)
Riboflavin, mg	1.4 (1.1–2.5)	1.4 (0.8–2.7)
Niacin, NE	25.8 (16.2–39.8)	23.0 (12.0–34.2)
Vitamin B6, mg	1.6 (1.0–2.7)	1.5 (0.9–2.5)
Folate, µg	307 (182–661)	336 (161–765)
Vitamin B12, µg	6.2 (2.7–16.1)	4.8 (2.0–16.9)
Vitamin C, mg	97 (31–243)	125 (33–312)
Calcium, mg	851 (497–1377)	766 (431–1379)
Phosphorus, mg	1302 (925–2151)	1221 (752–1836)
Potassium, mg	2884 (1990–4579)	2807 (1781–4175)
Iron, mg	9.9 (6.1–16.4)	8.9 (5.1–14.0)
Zinc, mg	9.7 (6.3–16.0)	9.2 (5.1–12.9)
Copper, mg	2.4 (0.7–6.3)	2.4 (0.8–5.9)
Iodine, µg	127 (64–274)	104 (38–252)
Selenium, µg	49.5 (25.4–90.6)	37.8 (21.4–81.2)

**Table 3:** Percentage of study population below (and above) the Nordic Nutritional Recommendations (NNR) Dietary Reference Values (DRV) for different macronutrients, dietary fiber and alcohol.

	NNR DRV	Men	Women	Total
		(*n* = 79)	(*n* = 78)	(*n* = 157)
Protein	RIR: 15–20 E%	18 * (30) **	21 (19)	19 (25)
	RIR: 1.1–1.3 g/kg BW	47 (28)	44 (37)	45 (32)
	AR ^$^: 0.66 g/kg BW	3 (97)	4 (96)	3 (97)
Carbohydrates	RIR: 45–60 E%	44 (5)	38 (3)	41 (4)
Dietary fiber	RI: ≥ 35 g/day (men), ≥ 25 g/day (women)	82	54	68
Fat	RIR: 25–40 E%	8 (27)	8 (23)	8 (25)
Saturated fat	RI: <10 E%	71	60	66
Mono-unsaturated fat	RIR: 10–20 E%	52 (1)	42 (3)	47 (2)
Poly-unsaturated fat	RIR: 5–10 E%	63 (3)	61 (1)	62 (2)
Alcohol	RI: <5 E%	56	45	50

* Below reference value, ** Above reference value, ^$^ Protein is the only macronutrient with an estimated Average Requirement (AR). RIR; Recommended Intake Range, RI; Reference Intake.

**Table 4:** Percentage of study population with intakes below the estimated Average Requirements (AR) for selected micronutrients.

	Reference Value	Men	Women	Total
		(*n* = 79)	(*n* = 78)	(*n* = 157)
Vitamin A	600 RE (men) 500 RE (women)	33	27	30
Thiamine	1.2 mg (men) 0.9 mg (women)	57	33	45
Riboflavin	1.4 mg (men) 1.1 mg (women)	38	26	32
Niacin	15 NE (men) 12 NE (women)	5	4	4
Vitamin B6	1.3 mg (men) 1.1 mg (women)	23	12	17
Folate	200 µg	10	13	11
Vitamin B12	1.4 µg	0	3	1
Vitamin C	60 mg (men) 50 mg (women)	25	13	19
Vitamin D	7.5 µg	76	83	80
Vitamin E	6 α-TE (men) 5 α-TE (women)	46	18	32
Calcium	500 mg	5	12	8
Phosphorus	450 mg	0	0	0
Iron	7 mg (men) 6 mg (women)	16	13	15
Zinc	6 mg (men) 5 mg (women)	3	5	4
Copper	0.7 mg	5	4	4
Iodine	100 µg	28	46	37
Selenium	35 mg (men) 30 mg (women)	25	33	27

**Table 5:** Potential determinants of nutrient intake.

	Youngest	Oldest	*p*-Value **	Married/Cohabitating	Living Alone	*p*-Value	Healthy Attitude	Non-Healthy Attitude	*p*-Value	Highest Energy to Bodyweight Ratio	Lowest Energy to Bodyweight Ratio	*p*-Value
Protein RIR: 1.1 g/kg	39.6 *	47.7	0.39	43.0	44.9	0.83	41.8	51.1	0.10	11.5	73.6	<0.001
Dietary fiber RI: ^$^ 25 & 35 g/day	56.3	73.4	0.05	67.4	61.2	0.57	57.0	78.7	0.28	49.2	77.8	<0.001
Vitamin D AR: 7.5 µg/day	83.3	75.4	0.31	74.4	83.7	0.21	70.9	89.3	0.02	72.1	82.0	0.18
Thiamine AR: ^$^ 0.9 & 1.2 mg/day	43.8	46.1	0.80	44.2	42.9	0.88	35.5	45.2	0.69	33.9	44.8	0.22
Iodine AR: 100 µg/day	35.4	36.9	0.87	37.2	36.7	0.95	38.4	34.0	0.66	29.5	43.1	0.11
Selenium AR: ^$^ 30 & 35 mg/day	20.8	36.9	0.07	22.1	36.7	0.07	24.4	34.0	0.03	26.2	29.2	0.71

* Percentage of individuals below the lower bound of the Recommended Intake Range (RIR), the Reference Intake (RI) or Average Requirement (AR). ** Chi-squared test for differences between groups. ^$^ Different reference values for men and women.

## Abbreviations

AMDR	Acceptable Macronutrient Distribution Range
AR	Average Requirement
EAR	Estimated Average Requirement
EFSA	European Food Safety Authority
g/kg BW	gram per kilo bodyweight
MUFA	Mono-Unsaturated Fatty Acids
NNR	Nordic Nutritional Recommendations
PUFA	Poly-Unsaturated Fatty Acids
SFA	Saturated Fatty Acids
RI	Reference Intake
RIR	Recommended Intake Range for macronutrients
